# Effects of mycophenolate mofetil on proliferation and mucin-5AC expression in human conjunctival goblet cells in vitro

**Published:** 2010-10-01

**Authors:** Hong He, Hui Ding, Aiping Liao, Qiong Liu, Jun Yang, Xingwu Zhong

**Affiliations:** 1 Zhongshan Ophthalmic Center and State Key Laboratory of Ophthalmology, Sun Yat-sen University, Guangzhou, China; 2 University of Houston, College of Optometry, Houston, TX

## Abstract

**Purpose:**

To investigate the effects of mycophenolate mofetil (MMF) on proliferation and mucin-5AC (*MUC5AC*) mRNA expression of normal human conjunctival goblet cells (CGCs) in vitro and to understand mechanisms of MMF in treatment of dry eye syndrome at molecular level.

**Methods:**

Purified human CGCs were treated with a series of graded concentrations of MMF after being confirmed by immunocytochemistry and flow cytometry. Proliferation and *MUC5AC* mRNA expression of CGCs were measured by Cell Count Kit-8 (CCK-8) and quantitative nested real-time reverse transcription polymerase chain reaction (QNRT-PCR at 24 h after treatment. The cell proliferation and *MUC5AC* mRNA expressiion were compared among different doses of MMF.

**Results:**

MMF induced a dose-dependent upregulation of *MUC5AC* mRNA expression (F=238.851, p<0.01) but a biphase effect on proliferation of the CGCs over 24 h of co-incubation. This biphase effect manifested as a dose-dependent increase in cell numbers with MMF from 0.25 to 2.5 ng/ml, an unchanged population of the cells from 2.5 to 10 ng/ml and a reduced population of the cells from 25 to 100 ng/ml.

**Conclusions:**

MMF exerts biphase effects on cell regeneration and upregulates *MUC5AC* mRNA expression in CGCs in vitro. It appears that the use of MMF at low concentrations is attractive in dry eye (DE) treatment.

## Introduction

Conjunctival goblet cells (CGCs) are essential for maintaining the integrity of the ocular surface. As highly specialized epithelial cells, CGCs are capable of producing multiple mucin proteins among which, Mucin-5AC (MUC5AC) is a high-molecular-weight glycoprotein and the major component of the mucous layer of the tear film. As an essential component of tear film, it acts as a barrier to protect the ocular surface from noxious stimulation and helps maintain the stability of tear film [1-4]. Because tear film instability is considered to be one of the key factors of dry eye (DE), CGCs and MUC5AC have drawn wide attention in DE studies. Impression cytology shows that patients with moderate to severe DE have a decreased number of CGCs. Conjunctival MUC5AC deficiency is also present in DE patients, and is associated with Sjogren syndrome (SS) and Stevens-Johnson syndrome [5-7].

It is now recognized that inflammation plays a key role in the pathogenesis of DE [8,9]. In animal models, DE is associated with T- and B-cell mediated inflammation [10,11]. In I kappa B zata gene-disrupted mice, infiltration of CD45R/B220(+) B and CD4(+) T cells in the ocular surface is usually associated with a reduced number of goblet cells [12,13]. The population of T and B cells with the pro-inflammatory cytokines increases in the lacrimal functional unit (LFU) of DE patients [8,10,14-17]. CGCs can be damaged by an activated inflammatory cascade. Both B and T cells migrate into LFU, produce inflammatory cytokines (such as IFN-γto) and therefore enhance the immune responses as evidenced by an increase in adhesion molecules produced by conjunctival tissue. Thus, interruption of such an inflammatory cascade by suppressing the activities of B and T cells maybe effective in protecting CGCs.

A variety of dry eye treatments have been focused on restoring the normal tear film, repairing damage of the ocular surface, and relieving the symptoms. Immunosuppressive agents have been widely investigated because they are capable of inhibiting the vicious circle of inflammation in DE. Cyclosporine A (CsA), an inhibitor of T cells, has been approved by the Food and Drug Administration for use in the treatment of the DE condition in humans [6,18]. Topical CsA is effective clinically, but still in limited use due to side effects such as eye hyperemia, stinging, burning, and transient blur vision [19]. Therefore, a clinically acceptable immunosuppressive should be effective with minimal side effects.

Mycophenolate mofetil (MMF) is an inhibitor to T and B lymphocytes, which has been reported to be effective in the treatment of transplant rejection and multiple autoimmune diseases. Unlike CsA, MMF does not interfere with IL-2 pathways. It suppresses the immunosuppressive system by reversibly inhibiting inosine-5-monophosphate dehydrogenase in the purine synthesis pathway [20]. Previous studies have reported that MMF is effective and safe in prolonging the survival of corneal transplants and in the treatment of ocular inflammation diseases. In addition, evidence also suggests two main superiorities of MMF compared with other immunosuppressive drugs, such as CsA: 1. lower side effects and better tolerability, and 2. a synergistic effect with other immunosuppressive drugs and corticosteroids [21-26]. Theoretically, MMF may be a potent immunosuppressive agent in the management of DE.

This study investigated effects of MMF on in vitro growth of human CGCs to understand the molecular mechanisms of MMF in the treatment of dry eye syndrome.

## Methods

### Isolation and culture of CGCs

Human CGCs were isolated as described previously [4]. In brief, normal human conjunctival tissues were obtained from patients who underwent periocular surgery after informed consent at the Zhongshan Ophthalmic Center. The procedure followed the ethical guidelines of the Declaration of Helsinki. Surgically excised tissues were immediately placed in D-Hanks solution consisting of 300 μg/ml penicillin-streptomycin. Three to four minced tissues explants were placed into one well of a six-well plate pre-coated with fetal bovine serum, and kept in a humidified incubator (95% air and 5% CO_2_ at 37 °C) for 15 min. Adherent explants were fed every two days with RPMI 1640 medium supplemented with 10% fetal bovine serum (FBS), 2 mM glutamine, and 100 μg/ml penicillin-streptomycin, and grown under routine culture conditions (95% air and 5% CO_2_ at 37 °C). After 3–5 days, the tissue explants were removed to avoid contamination by nongoblet cells. If even one tissue explant displayed connective tissue morphology, the whole well was discarded. Nongoblet cells were discarded using a conventional plastic cell scraper. A further purification was performed by enzymatic digestion method. Two to five passage goblet cells (purity over 98.00%) were used in all experiments.

### Immunocytochemistry

The cultured cells were examined for the presence of cytokeratins 4 and 7 (CK-4, CK-7)and for MUC5AC. Passage cells were seeded on coverslips at a density of 1×10^4^ and incubated at 37 °C in humid atmosphere of 5% CO_2_/95% O_2._ After three days, confluent CGCs were fixed with 4% paraformaldehyde for 15 min, washed with PBS three times, permeabilized in 1% Triton X-100 for 20 min, washed three times, and blocked with 1% BSA for 30 min. Then, fixed and permeabilized cells were incubated with the following primary antibodies: mouse anti-human cytokeratin 7 (1:200 in PBS; Abcam, Cambridge, UK), rabbit anti-human MUC5AC (1:100 in PBS; Abcam), and mouse anti-human cytokeratin 4 (1:200 in PBS; Abcam). The secondary antibodies were fluorescein isothiocyanate (FITC)- and tetramethylrhodamine isothiocyanate-conjugated anti-mouse and anti-rabbit antibodies (1:50 in PBS; Abcam). The primary antibodies were incubated for 1 h at room temperature after being maintained overnight at 4 °C. The second antibodies were incubated for 2 h at room temperature. Negative control staining was performed by omitting the primary antibodies. The cells were viewed with a confocal microscope (Carl Zeiss, Gottingen, Germany).

### Flow cytometry analysis of the purity of the CGCs in culture

The percentage of cells found to be immunoreactive for cytokeratin 7 (CK-7), a specific marker of goblet cells, was evaluated using flow cytometry with an Elite flow cytometer (Beckman-Coulter, Brea, CA). Briefly, purified cells were pretreated with the Fix & Perm cell permeabilization buffer (eBioscience, San Diego, CA). The monoclonal antibody against CK-7 (1:100 in PBS; Abcam) was incubated at 4 °C for 30 min. The FITC-labeled second antibody (1:200 in PBS; Caltag, Fremont, CA) was incubated at 4 °C for 30 min while protected from light.

### In vitro proliferation assays

Cell proliferation assays were performed using a Cell Counting Kit-8 (CCK-8; Donjindo, Kumamoto, Japan) according to the manufacturer’s instructions. Purified CGCs (1×10^5^/well) were cultured in flat-bottom 96-well microtiter plates with the plating medium. One day later, the medium was refed with the growth medium containing the MMF (Roche, Mannheim, Germany), or the growth medium without agent (control group). The concentrations of MMF in the medium ranged from 0.25 to 100 ng/ml. After 24 h, the cells in each well were measured as the absorbance (450 nm). All measurements were performed in sextuplicate. Results were expressesd as mean of A450±SD. The percent of CGCs proliferation was calculated as follows: proliferating cell (%)=[(Ae-Ab)/(Ac-Ab)]*100%, where Ae, Ab and Ac are A450 of experimental, blank and control group, respectively.

### Quantitative nested real-time reverse transcription polymerase chain reaction (QNRT–PCR)

#### Total RNA isolation and cDNA synthesis

Purified cells were seeded in 12-well plates with the growth medium, and grew to 75% confluence. Cells were stimulated without (control) or with MMF (0.25 ng/ml, 2.5 ng/ml, and 25 ng/ml), and incubated for 24 h. For RNA extraction, 1 ml Trizol (Invitrogen, Carlsbad, CA) were added to CGCs sample, and were then gradually processed using 0.2 ml chloroform (Sigma-Aldrich, St. Louis, MO) and avantin (Sigma-Aldrich). After centrifugation, supernatant was removed. Extracted RNA pellet was then washed with 75% ethanol (Sigma-Aldrich) and centrifuged at 7,500 r/min for 5 min at 4 °C. After vacuum desiccation (5–10 min), the extracted RNA specimen was resuspended in diethyl pyrocarbonate- (DEPC) treated water and then stored at −80 °C until it was used. Reverse transcription of RNA was performed with ABI Geneamp 9700 PCR systems (Applied Biosystems, Foster City, CA) with cDNA synthesized from 4 μg of total RNA.

#### Two steps of quantitative nested real-time transcription polymerase chain reaction (QNRT-PCR)

QNRT–PCR consists of two consecutive PCR amplification steps. In the first step, a 10 μl mixture of the PCR solution containing 10 mM Tris-HCl (pH 8.0), 50 mM KCl, 1.5 mM MgCl_2_, 10 mM each of deoxynucleoside triphosphate mixture, 3 U of Taq DNA polymerase, and 10 pM (each) of the outer forward or reverse primers were prepared. Four μl of the cDNA specimen as the template was amplified by using the outer forward (F-1) or reverse (R-1) primer, under the same assay conditions as in the conventional single PCR (Table 1 and Table 2). For the second step, a pair of inner primers for *MUC5AC* (TaqMan forward primer [TqMn-F] and TaqMan reverse primer [TqMn-R]) were prepared (Table 1). In addition, two specific TaqMan probes, which were labeled with the fluorescent reporter dye 6-carboxyfluorescein (FAM), were prepared (Table 1). As the template, 5 μl of the first step PCR product at the second step was added to the PCR solution mixture (each total reaction volume was 50 μl). This preparation was subjected to the protocol shown in Table 2, and the procedure used the ABI Geneamp 9700 PCR system (Applied Biosystems).

**Table 1:** Sequences of primer and TaqMan probes for PCR assays.

**Object**	**Type**	**Sequence**
QNRT–PCR assay		
First-step PCR	outer forward primer	5′-TGACGGACCTGGATGTGGT-3′
** **	outer reverse primer	5′-TGTCATTCCCGTAGCAGTAGGA-3′
Second-step PCR	TaqMan forward primer	5′-TGCGTCCCACGACATCTG-3′
** **	TaqMan reverse primer	5′-CAGGTGAATGGGCACATGTG-3
** **	TaqMan probe-mutation-FAM	5′-FAM-ATCGATTGGAGAGGCCGGACCG-TAMRA-3′
Internal control	Human-β-actin forward primer	5′-GCATGGGTCAGAAGGATTCCT-3′
** **	Human-β-actin reverse primer	5′-TCGTCCCAGTTGGTGACGAT-3′
** **	TaqMan probe-mutation-FAM	5′-FAM-CCTCACCCTGAAGTACCCCATCGAGC-TAMRA-3′

**Table 2:** PCR assay conditions.

** **	**Value**	
**PCR assay conditions**	**First-step PCR**	**Second-step PCR**
QNRT–PCR		
Initial denature	93.0 °C, 5 min	** **
Amplification		
Denature	93.0 °C, 30 s	** **
Annealing	55.0 °C, 45 s	** **
Extension	72.0 °C, 45 s	** **
Final extension	72.0 °C, 7 min	** **
Incubation	** **	93.0 °C, 3 min
Initial denature	** **	95.0 °C, 10 min
Amplification		
Denature	** **	93.0 °C, 30 s
Annealing-extension	** **	55.0 °C, 45 s

## Results

### Characterization and purity of CGCs in culture

**Figure 1:**
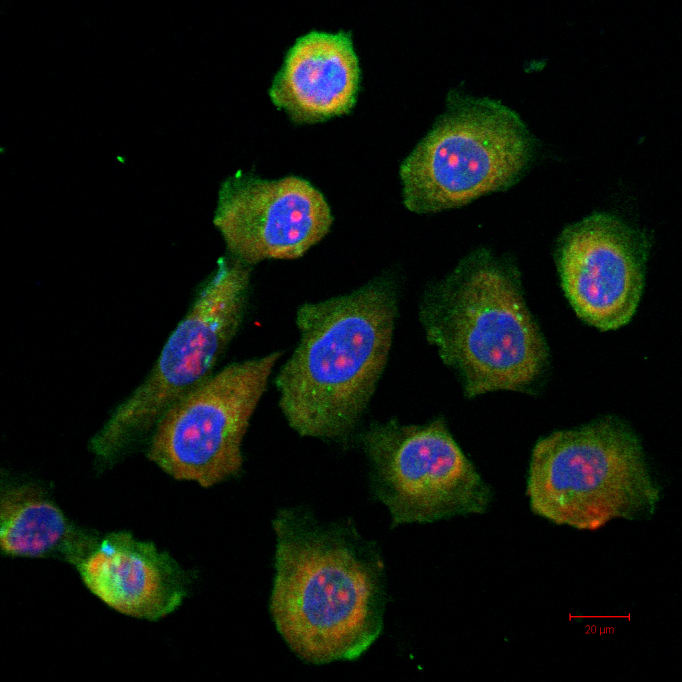
Photomicrographs of CGCs in vitro. Double immunolabeling for CK-7 and MUC5AC indicate the presence of CK-7 (shown in green) and MUC5AC (shown in red) in the same cell in culture. The scale bar represents 20 μm.

**Figure 2:**
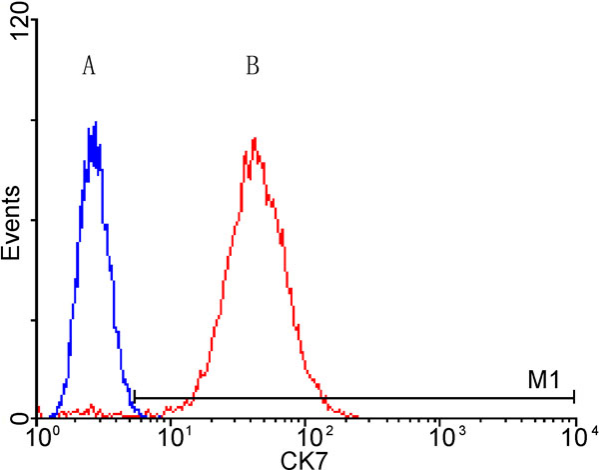
Flow cytometry analysis of CK-7 expression. **A**: negative control. **B**: with antibody against CK-7. Numenator histogram gated events: 9,806. Denominator histogram gated events: 10,000% Gated: 98.06%.

Double immunolabeling (CK-7 and MUC5AC) and confocal microscopy analysis were used to identify cultured CGCs. It was found that passaged cells stained intensely for CK-7 and MUC5AC, which were well established to be specific markers of CGCs, while they did not stain for CK-4. CK-7 and MUC5AC often colocalized in the same passaged cell (Figure 1). CK-7, as an intermediate filament, was located in the cytoplasm. MUC5AC, located within the secretory granules, was shown under punctate staining. To further evaluate the purity of purified cells, flow cytometry was used. The purity of CGCs, shown as the percentage of CK-7(+) cells, was indicated to be over 98.06% (Figure 2).

### Effect of MMF on proliferation of human CGCs in culture

**Figure 3:**
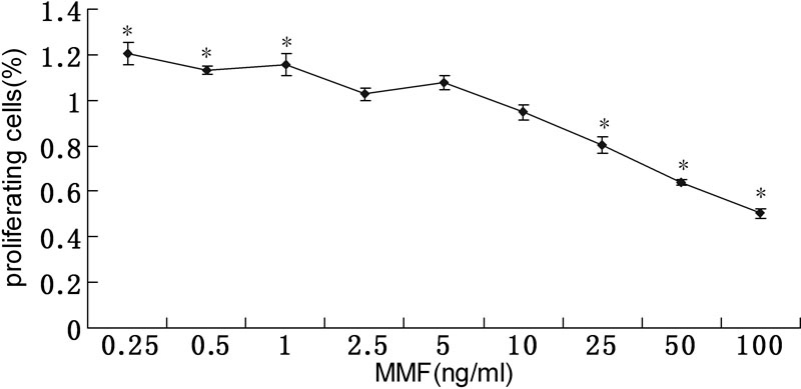
Biphase effects of MMF on proliferation in CGCs in vitro. CGCs were incubated for 24 h with increasing concentrations of MMF. Three independent experiments were analyzed by CCK-8, and means±SD are shown in the graph. *p<0.01 versus control by ANOVA.

MMF exerted biphase effects on the proliferation of human CGCs (Figure 3). After intervention with MMF for 24 h, it was shown that MMF, within the range of 0.25 ng/ml-1 ng/ml, significantly increased CGC proliferation (p<0.01). However, at higher concentrations (25 ng/ml, 50 ng/ml, and 100 ng/ml), MMF decreased CGCs proliferation (p<0.01). In addition, CCK-8 also showed that MMF (2.5 ng/ml, 50 ng/ml, and 10 ng/ml) had no effect on the proliferation of CGCs (p*>*0.01). The lowest concentrations required to promote and/or inhibit cell viability were 0.25 ng/ml and/or 25 ng/ml, respectively, while the lowest concentration of MMF to have no cytotoxic effect was 2.5 ng/ml.

### MMF increases *MUC5AC* mRNA expression in human CGCs in culture

**Figure 4:**
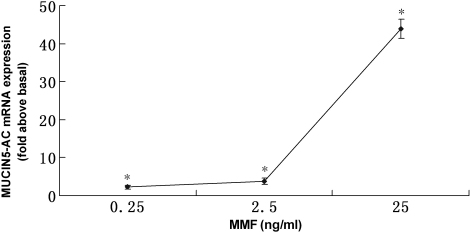
Enhancing effect of *MUC5AC* mRNA expression in CGCs in vitro after 24 h exposure to increasing concentrations of MMF. Three independent experiments were analyzed by QNRT–PCR, and means±SD are shown in the graph. *p<0.01 versus control by ANOVA.

MMF significantly increased *MUC5AC* mRNA expression (Figure 4) in a dose dependent manner. At concentrations of 0.25, 2.5, and 25 ng/ml, MMF significantly increased *MUC5AC* mRNA expression by 2.3±0.5, 3.8±0.8, and 43.9±2.6 times more than the control group respectively (p<0.01). The main peak of expression appears at the concentration of 25 ng/ml.

## Discussion

In this present study, MMF demonstrates a biphase effect on CGCs proliferation and an upregulation of *MUC5AC* mRNA expression, suggesting that an optimal dose is one of the important factors determining the effectiveness in clinical use of MMF. Dry eye syndrome is an inflammatory process triggered by multiple factors and inhibition of this process is critical to relieve the symptoms and signs for DE patients. Since both T and B cells are involved in the inflammatory process of DE, MMF which is an inhibitor for T and B cells with relatively mild adverse responses has been investigated as a potential agent for treatment of DE patients. Interestingly, a enhancing effect of MMF on proliferation of CGCs at lower concentrations is demonstrated in this present study. At a dose of 0.25, 0.5 and 1 ng/ml, MMF increases cell viability by 15.6%, 13%, and 20.6%, respectively, when compared to the control group. As the number and integrity of goblet cells are associated with the outcome of DE, the capability of MMF in stimulating proliferation of CGCs may provide a better alternative to current DE therapies if it is proven in vivo studies. A dose of MMF at 100, 50 or 25 ng/ml for 24 h can reduce cell viability by 49.7%, 36.3% or 19.6%, respectively, compared to the control group. This finding is consistent with studies using MMF (5–50 ng/ml) on other cells such as lymphocytes, monocytes, vascular smooth muscle cells, fibroblasts, and human intrahepatic biliary epithelial cells [20,27-30].

Previous studies confirm that the cytostatic effect of MMF on lymphocytes is to deplet intracellular guanosine nucleosides by reversibly inhibiting inosione-5-monophosphate dehydrogenase (IMPDH) in the de novo purine synthesis pathway [20,31-34]. However, mechanisms of MMF involved in the proliferation-enhancement of cells are not clear. In general, there are two pathways for biosynthesis of purine in cells – the de novo pathway and the salvage pathway. Goblet cells of the eye may rely on these two pathways to form purines for synthesis of DNA and RNA of the cells. Since IMPDH is not involved in the salvage pathway, the proliferation enhancing effect may be due to the increased purine synthesis by the salvage pathway to compensate absence of the de novo pathway. Goblet cells, which synthesize and secretes MUC5AC, are located not only in conjunctiva but also in other epithelial tissues. Little is known about the genes associated with MUC5AC production in CGCs, however, SAM-pointed domain–containing Ets-like factor (SPDEF) has been demonstrated to be the key genetic switch in pulmonary and intestinal goblet cells. It has been shown that SPDEF can induce mRNA expression of *MUC5AC* in progenitors of goblet cells with no associated proliferation of goblet cells [35] in the respiratory tract, known as the progenitors of goblet cells. This result is similar to a mismatch between *MUC5AC* mRNA expression and proliferation of the associated CGCs under treatment with higher concentrations of MMF in the present study. This mismatch indicates that an increased secretion of mucin in tear film could be due to an enhanced function of the individual CGCs rather than the increased number of the cells.

The minimum effective concentration (MEC) of MMF in the control of auto-immune response for DE has been included in the concentrations used in the present study based on previous in vitro and in vivo studies [36,37]. In addition, the doses of MMF used in this present study are similar to those currently recommended in the Spare-the-Nephron trial where patients were treated with MMF and sirolimus (an immunosuppressive drug produced by the actinomycete *Streptomyces hygroscopicus*) [37]. In the Spare-the-Nephron trial, an oral dose (1–1.5 g/kg) of MMF twice daily produces a plasma concentration varying from 5 to 10 ng/ml which can effectively suppress the lympholeukocytes. Therefore, MMF at doses from 0.5 ng.ml to 10 ng/ml in the present study are expected to effectively inhibit the lymphocytes involved in the inflammation of DE, in addition to its protential to enhance the protective role of the CGCs. Furthermore, MMF at 0.25 to 0.5 ng/ml appears to be the optimal dose in the treatment of DE with respect to the characteristics of minimum effective dose, great human CGCs proliferation enhancing effect, and significant upregulation of *MUC5AC* mRNA expression. However, whether MMF can suppress the immune system and provide protective therapy for dry eye patients at this range of doses still needs to be investigated in vivo.

In conclusion, this study demonstrates that MMF exerts biphase effects on proliferation and upregulates *MUC5AC* mRNA expression of CGCs. It appears that MMF adjusted at an optimal concentration has a potential effect to control the inflammation and enhance the function of goblet cells for patients having dry eye syndrome.

## References

[1] Mantelli F Argueso P Functions of ocular surface mucins in health and disease. Curr Opin Allergy Clin Immunol 2008 8 477 83 18769205 10.1097/ACI.0b013e32830e6b04 PMC2666617

[2] Gipson IK Argueso P Role of mucins in the function of the corneal and conjunctival epithelia. Int Rev Cytol 2003 231 1 49 14713002 10.1016/s0074-7696(03)31001-0

[3] Foulks GN The correlation between the tear film lipid layer and DE disease. Surv Ophthalmol 2007 52 369 74 17574063 10.1016/j.survophthal.2007.04.009

[4] Bron AJ Tiffany JM Gouveia SM Yokoi N Voon LW Functional aspects of the tear film lipid layer. Exp Eye Res 2004 78 347 60 15106912 10.1016/j.exer.2003.09.019

[5] Ramamoorthy P Nichols JJ Mucins in contact lens wear and DE conditions. Optom Vis Sci 2008 85 631 42 18677222 10.1097/OPX.0b013e3181819f25

[6] Kunert KS Tisdale AS Gipson IK Goblet cell numbers and epithelial proliferation in the conjunctiva of patients with DE syndrome treated with cyclosporine. Arch Ophthalmol 2002 120 330 7 11879137 10.1001/archopht.120.3.330

[7] Murube J Rivas L Biopsy of the conjunctiva in DE patients establishes a correlation between squamous metaplasia and DE clinical severity. Eur J Ophthalmol 2003 13 246 56 12747645 10.1177/112067210301300302

[8] Pepose JS Akata RF Pflugfelder SC Voigt W Mononuclear cell phenotypes and immunoglobulin gene rearrangements in lacrimal gland biopsies from patients with Sjogren’s syndrome. Ophthalmology 1990 97 1599 605 1965021 10.1016/s0161-6420(90)32372-2

[9] Williamson J Gibson AA Wilson T Forrester JV Whaley K Dick WC Histology of the lacrimal gland in keratoconjunctivitis sicca. Br J Ophthalmol 1973 57 852 8 4785251 10.1136/bjo.57.11.852 PMC1215232

[10] Hassan AS Clouthier SG Ferrara JL Stepan A Mian SI Ahmad AZ Elner VM Lacrimal gland involvement in graft-versus-host disease: a murine model. Invest Ophthalmol Vis Sci 2005 46 2692 7 16043840 10.1167/iovs.05-0040

[11] van Blokland SC Versnel MA Pathogenesis of Sjogren’s syndrome: characteristics of different mouse models for autoimmune exocrinopathy. Clin Immunol 2002 103 111 24 12027416 10.1006/clim.2002.5189

[12] Ueta M Hamuro J Yamamoto M Kaseda K Akira S Kinoshita S Spontaneous ocular surface inflammation and goblet cell disappearance in I kappa B zeta gene-disrupted mice. Invest Ophthalmol Vis Sci 2005 46 579 88 15671285 10.1167/iovs.04-1055

[13] Niederkorn JY Stern ME Pflugfelder SC De Paiva CS Corrales RM Gao J Siemasko K Desiccating stress induces T cell-mediated Sjogren’s Syndrome-like lacrimal keratoconjunctivitis. J Immunol 2006 176 3950 7 16547229 10.4049/jimmunol.176.7.3950

[14] Zhan H Towler HM Calder VL The immunomodulatory role of human conjunctival epithelial cells. Invest Ophthalmol Vis Sci 2003 44 3906 10 12939308 10.1167/iovs.02-0665

[15] Pflugfelder SC Huang AJ Feuer W Chuchovski PT Pereira IC Tseng SC Conjunctival cytologic features of primary Sjogren’s syndrome. Ophthalmology 1990 97 985 91 1698273 10.1016/s0161-6420(90)32478-8

[16] Gulati A Sacchetti M Bonini S Dana R Chemokine receptor CCR5 expression in conjunctival epithelium of patients with DE syndrome. Arch Ophthalmol 2006 124 710 6 16682594 10.1001/archopht.124.5.710

[17] Stern ME Gao J Schwalb TA Ngo M Tieu DD Chan CC Reis BL Whitcup SM Thompson D Smith JA Conjunctival T-cell subpopulations in Sjogren’s and non-Sjogren’s patients with DE. Invest Ophthalmol Vis Sci 2002 43 2609 14 12147592

[18] Wilson SE Perry HD Long-term resolution of chronic DE symptoms and signs after topical cyclosporine treatment. Ophthalmology 2007 114 76 9 17070588 10.1016/j.ophtha.2006.05.077

[19] Barber LD Pflugfelder SC Tauber J Foulks GN Phase III safety evaluation of cyclosporine 0.1% ophthalmic emulsion administered twice daily to DE disease patients for up to 3 years. Ophthalmology 2005 112 1790 4 16102833 10.1016/j.ophtha.2005.05.013

[20] Allison AC Eugui EM Mycophenolate mofetil and its mechanisms of action. Immunopharmacology 2000 47 85 118 10878285 10.1016/s0162-3109(00)00188-0

[21] Chanaud NP Vistica BP Eugui E Nussenblatt RB Allison AC Gery I Inhibition of experimental autoimmune uveoretinitis by mycophenolate mofetil, an inhibitor of purine metabolism. Exp Eye Res 1995 61 429 34 8549684 10.1016/s0014-4835(05)80138-1

[22] Baltatzis S Tufail F Yu EN Vredeveld CM Foster SM Mycophenolate mofetil as an immunomodulatory agent in the treatment of chronic ocular inflammatory disorders. Ophthalmology 2003 110 1061 5 12750115 10.1016/S0161-6420(03)00092-7

[23] Daniel E Thorne JE Newcomb CW Pujari SS Kacmaz RO Levy-Clarke GA Nussenblatt RB Rosenbaum JT Suhler EB Foster CS Jabs DA Kempen JH Mycophenolate mofetil for ocular inflammation. Am J Ophthalmol 2010 149 423 32 20042178 10.1016/j.ajo.2009.09.026 PMC2826576

[24] Sobrin L Christen W Foster CS Mycophenolate mofetil after methotrexate failure or intolerance in the treatment of scleritis and uveitis. Ophthalmology 2008 115 1416 21 18221998 10.1016/j.ophtha.2007.12.011

[25] Liang L Sheha H Tseng SC Long-term outcomes of keratolimbal allograft for total limbal stem cell deficiency using combined immunosuppressive agents and correction of ocular surface deficits. Arch Ophthalmol 2009 127 1428 34 19901207 10.1001/archophthalmol.2009.263

[26] Anil Kumar MS Irfan Saeed M Ranganna K Malat G Sustento-Reodica N Kumar AM Meyers WC Comparison of four different immunosuppression protocols without long-term steroid therapy in kidney recipients monitored by surveillance biopsy: five-year outcomes. Transpl Immunol 2008 20 32 42 18773960 10.1016/j.trim.2008.08.005

[27] Gregory CR Pratt RE Huie P Shorthouse R Dzau VJ Billingham ME Morris RE Effects of treatment with cyclosporine, FK 506, rapamycin, mycophenolic acid, or deoxyspergualin on vascular muscle proliferation in vitro and in vivo. Transplant Proc 1993 25 770 1 7679842

[28] Eugui EM Almquist SJ Muller CD Allison AC Lymphocyte-selective cytostatic and immunosuppressive effects of mycophenolic acid in vitro: role of deoxyguanosine nucleotide depletion. Scand J Immunol 1991 33 161 73 1826793 10.1111/j.1365-3083.1991.tb03746.x

[29] Liu C Frilling A Dahmen U Broelsch CE Gerken G Treichel U Cyclosporine A FK-506, 40–0-[2-hydroxyethyl]rapamycin and mycophenolate mofetil inhibit proliferation of human intrahepatic biliary epithelial cells in vitro. World J Gastroenterol 2005 11 7602 5 16437685 10.3748/wjg.v11.i48.7602 PMC4727236

[30] Morath C Zeier M Review of the antiproliferative properties of mycophenolate mofetil in non-immune cells. Int J Clin Pharmacol Ther 2003 41 465 9 14703952 10.5414/cpp41465

[31] Allison AC Hovi T Watts RW Webster AD The role of de novo purine synthesis in lymphocyte transformation. Ciba Found Symp 1977 48 207 24 415850 10.1002/9780470720301.ch13

[32] Morris RE Hoyt EG Murphy MP Eugui EM Allison AC Mycophenolic acid morpholinoethylester (RS-61443) is a new immunosuppressant that prevents and halts heart allograft rejection by selective inhibition of T- and B-cell purine synthesis. Transplant Proc 1990 22 1659 62 2389428

[33] Allison AC Eugui EM Mechanisms of action of mycophenolate mofetil in preventing acute and chronic allograft rejection. Transplantation 2005 80 S181 90 16251851 10.1097/01.tp.0000186390.10150.66

[34] Yashima Y Ohgane T Pharmacological profiles of mycophenolate mofetil (CellCept), a new immunosuppressive agent. Nippon Yakurigaku Zasshi 2001 117 131 7 11233304 10.1254/fpj.117.131

[35] Chen G Korfhagen TR Xu Y SPDEF is required for mouse pulmonary goblet cell differentiation and regulates a network of genes associated with mucus production. J Clin Invest 2009 119 2914 24 19759516 10.1172/JCI39731 PMC2752084

[36] Carr SF Papp E Wu JC Natsumeda Y Characterization of human type I and type II IMP dehydrogenases. J Biol Chem 1993 268 27286 90 7903306

[37] Pearson TC Patel A Scandling J Shidban H Weir M Patel D Mulgaonkar S Final Results of the Spare the Nephron Trial (Cni Withdrawal Trial): 629. Transplantation 2008 86 220 1

